# Investigating the biological response of human mesenchymal stem cells to titanium surfaces

**DOI:** 10.1186/s13018-014-0135-y

**Published:** 2014-12-12

**Authors:** Matthew J German, Charles Osei-Bempong, Callie A Knuth, David J Deehan, Rachel A Oldershaw

**Affiliations:** Centre for Oral Health Research, School of Dental Sciences, Faculty of Medical Sciences, Newcastle University, Newcastle upon Tyne, NE2 4BW UK; Institute of Genetic Medicine, Faculty of Medical Sciences, Newcastle University, International Centre for Life, Times Square, Newcastle upon Tyne, NE1 4EP UK; North East England Stem Cell Institute, Faculty of Medical Sciences, Newcastle University, International Centre for Life, Times Square, Newcastle upon Tyne, NE1 4EP UK; Department of Orthopaedics, Freeman Hospital, Newcastle upon Tyne NHS Hospitals Foundation Trust, Freeman Road, High Heaton, Newcastle upon Tyne, NE7 7DN UK; Department of Musculoskeletal Biology, Institute of Ageing and Chronic Disease, Faculty of Health and Life Sciences, The University of Liverpool, Leahurst Campus, Chester High Road, Neston, CH64 7TE UK

**Keywords:** Titanium screws, Tissue engineering, Mesenchymal stem cells, Biocompatibility

## Abstract

**Background:**

We have investigated the behaviour of a newly characterised population of haemarthrosis fluid-derived human mesenchymal stem cells (HF-hMSCs) with titanium (Ti) surfaces.

**Methods:**

HF-hMSCs were seeded onto round cannulated interference (RCI; Smith and Nephew) screws or control Ti discs and cultured under pro-osteogenic conditions.

**Results:**

Electron microscopy showed the attachment and spreading of HF-hMSCs across both Ti surfaces during the early stages of osteogenic culture; however, cells were exclusively localised to the basal regions within the vertex of the Ti screws. In the later stages of culture, an osteoid matrix was deposited on the Ti surfaces with progressive culture expansion and matrix deposition up the sides and the top of the Ti Screws. Quantification of cellular content revealed a significantly higher number of cells within the Ti screw cultures; however, there was no difference in the cellular health. Conversely, alizarin red staining used as both a qualitative and quantitative measure of matrix calcification was significantly increased in Ti disc cultures compared to those of Ti screws.

**Conclusions:**

Our results suggest that the gross topography of the metal implant is able to create microenvironment niches that have an influence on cellular behaviour. These results have implications for the design of advanced tissue engineering strategies that seek to use cellular material to enhance biological remodelling and healing following tissue reconstruction.

## Background

The use of inert material implants has provided the mainstay of dental, maxillofacial and orthopaedic reconstructive surgeries for decades [1]. Titanium (Ti) is one of the most common metals chosen for the manufacture of these medical devices with its chemistry providing high specific strength, a low Young’s modulus of elasticity that reduces the impact of peri-implant bone resorption, a high capacity for connecting to bone and soft tissues and the formation of a titanium oxide surface layer that supports biocompatibility and resistance to corrosion [1-5]. Because of these properties, Ti is often regarded as a more advantageous implant material, particularly for bone engineering, when compared to other metals such as cobalt, nickel, chromium and their respective alloys [2-7].

In recent years biomedical research has sought to develop more advanced tissue engineering strategies that augment the clinical outcomes of reconstructive surgeries, typically through stimulation of the biological tissue response to healing [1,8]. These approaches, mainly within the development stage at present, will often take the form of a cellular component (e.g. stem cells, progenitor cells or moderately expanded, mature, functional cells) combined with a biodegradable scaffold component that can provide a physical support for the cells as well as control physiologic behaviours such as differentiation (e.g. poly(lactic-*co*-glycolic acid) (PLGA); or poly-ε-caprolactone (PCL)) [9,10].

As a clinical exemplar of this evolution in surgical technologies, the focus of our research is the attachment of soft ligament tissue to bone and in particular the reconstruction of anterior cruciate ligament (ACL) following traumatic rupture. In this procedure, a Ti metal screw is used to fix pseudo-ligament tissue (typically taken from the hamstring tendon) within juxta-endosteal bony tunnels drilled at the anatomical anchor sites of the native ACL [11,12]. Whilst the screw is able to confer resistance to moderate biomechanical loading, the slow rate of biological tissue remodelling that ultimately leads to the osseous integration of the soft tissue graft can delay healing for up to 12 months post-surgery [13,14]. Revision surgery as a result of graft failure occurs within approximately 20% of cases and the prolonged period of joint destabilisation risks further meniscal and chondral damage in the short term as well as increasing the incidence of chronic degenerative joint diseases such as osteoarthritis [15,16].

We are seeking to develop a tissue engineering strategy to be used as an adjunct to conventional ACL reconstruction to improve the biological response post-surgery by enhancing and accelerating the osseous integration of the graft tissue. In previous work we have identified a novel population of osteogenic human mesenchymal stem cells derived from haemarthrosis fluid, the intra-articular bleed that is aspirated from the joint space during the acute phase of injury [17,18]. An investigation of the performance of these cells (termed heamarthrosis fluid-derived human mesenchymal stem cell (HF-hMSCs)) with a biomaterial capable of facilitating cell delivery and retention at the site of surgery has demonstrated the attachment, spreading and osteogenic differentiation on microparticles manufactured from PLGA [19].

The aim of this study is to investigate the behaviour of HF-hMSCs in response to titanium screws that are used to fix the soft tissue graft during standard ACL reconstruction. Here we report our initial findings comparing the attachment, proliferation and matrix mineralisation potential of HF-hMSCs in response to Ti discs and Ti RCI (Smith and Nephew) screws.

## Materials and methods

### Measurements of Ti surfaces

#### Ti discs and Ti screws

Ti discs (Grade 5, Ti6Al4V) were prepared by cutting 1-cm diameter rods to a length of 0.5 cm with a diamond saw. RCI Smith and Nephew screws (Grade 5, Ti6Al4V) were prepared by cutting the head and tapered end from each screw and sectioning lengthways using a diamond saw [20,21].

#### Roughness measurement

All of the roughness measurements were conducted using a stylus profilometer (Mitutoyo Surftest SV-2000 Mitutoyo, Halifax, UK) with dedicated analysis software (Surfpak- SV V1.600). A 5-μm radius diamond cone stylus tip was used to analyse the surface held at 90° to the surface with a contact force of 4 mN and a maximum height range of 800 μm. To measure roughness, a representative disc and screw specimen were selected. For the disc specimen, five randomly selected line scans were measured, with an evaluation length of 0.4 mm (5 sampling lengths of 0.08 mm). For the screw specimen, to measure the roughness in between the rakes, the specimen was held at 90° to the direction of travel of the profilometer, so that the stylus could sample the surface without making contact with a rake. A profile was measured between five successive rakes with an evaluation length of 0.4 mm (5 sampling lengths of 0.08 mm) used. All profiles were Gaussian filtered and baseline curvature was removed using the automatic software routine within the control software. As an estimate of roughness (Ra), the number average roughness was used.

#### Calculation of surface areas of Ti surfaces

The surface area of Ti discs exposed to cells was constant for all of the discs used within the study and was calculated as the surface area of a cylinder minus the area of the base:

$$ {\varGamma}_{\mathrm{d}}\kern0.5em =\kern0.5em \uppi {r}^{\wedge }2\kern0.5em +\kern0.5em 2\uppi rh $$

where Γ_d_ = the exposed surface area of the disc; *r* = the radius of the disc, 0.5 cm; *h* = the height of disc = 0.5 cm.

Using a diamond saw, the head and tapered end of each Ti RCI screw was removed to give a constant thread section that was sectioned lengthways to allow for stable placement inside each well of the cell culture cluster plate. The surface area of the Ti screw exposed to the cells was calculated using the equation derived by Rammer and Zelinka [22]:

$$ \frac{\varGamma_{\mathrm{s}}\kern0.5em =\kern0.5em {\varGamma}_{\mathrm{c}}\kern0.5em +\kern0.5em 2\pi \left(\frac{h}{\rho}\right)\left[\left({r}_t\kern0.5em +\kern0.5em {r}_c\right)\sqrt{{\left({r}_t\kern0.5em +\kern0.5em {r}_c\right)}^2\kern0.5em +\kern0.5em \left(\frac{{t_{\mathrm{w}}}^2}{4}\right)}\kern0.5em -\kern0.5em {r}_c{t}_{\mathrm{w}}\right]}{2} $$

where Γ_s_ = the exposed surface area of the screw; Γ_c_ is the core surface area; *h* is length of the screw threaded section, *ρ* is the distance between adjacent thread crests, *r*_t_ is the thread radius, *r*_c_ is the core radius and *t*_w_ is the thread width at the core diameter.

### Cell culture

#### Acquisition of cellular material and derivation of human mesenchymal stem cell lines from haemarthrosis fluid

This study was carried out with full approval from Newcastle and North Tyneside 2 Research Ethics Committee and Newcastle upon Tyne Hospitals NHS Foundation Trust R&D. HF was aspirated from consented patients presenting at a clinic with acute knee injury (*N* = 3 patient samples were used; 33-year-old male, 54-year-old female, 64-year-old male).

Human mesenchymal stem cells (HF-hMSCs) were derived as previously described [17-19]. Mononuclear cells were isolated by Ficoll® gradient centrifugation (700 × *g*, 20 min), collected by centrifugation (90,000 × *g*, 3 min) and seeded into T-75 culture flasks in MSC medium (alpha minimum essential medium (αMEM), 10% (*v*/*v*) foetal bovine serum (FBS), 5 ng/ml fibroblast growth factor-2 (FGF2)). Non-adherent cells were removed from culture 3 days post-seeding, and fresh MSC medium was added. HF-hMSCs were passaged upon confluence at a ratio of 1:3 and used in experiments at passage 2.

#### Osteogenic differentiation of haemarthrosis fluid-derived mesenchymal stem cells

Ti discs or Ti screws were polished, autoclaved twice and placed in individual wells of a 12-well cell culture cluster plate. HF-hMSCs were seeded in 1-ml volumes in MSC medium at a cell density of 2.5 × 10^4^ cells/cm^2^ Ti surface. Twenty-four hours post-seeding, the Ti discs and screws were transferred using aseptic technique into new wells of a fresh cell culture cluster plate, and the culture medium was replaced with osteogenic medium (αMEM, 10% (*v*/*v*) FBS, 20 ng/ml bone morphogenetic protein-2 (BMP2)) [19].

### Cell culture analysis

#### Scanning electron microscopy

Ti disc and Ti screw samples were rinsed with PBS and fixed with 2% (*w*/*v*) glutaraldehyde overnight at 4°C. Samples were then dehydrated through a graded series of ethanol (25% (*v*/*v*), 50% (*v*/*v*), 75% (*v*/*v*), 1 × 30 each; 100% (*v*/*v*) 2 × 1 h each before being mounted onto carbon tabs and sputter coated with 15 nm of gold. Microscopy was performed using a Cambridge Stereoscan 240 scanning electron microscope.

#### Measurement of cell content within cultures

The amount of deoxyribonucleic acid (DNA) within Ti disc and Ti screw cultures was measured as a means of quantifying the cellular content of the cultures. Ti discs and Ti screws were transferred to empty wells of the 24-well cell cluster plate and rinsed twice with Dulbecco’s phosphate-buffered saline (DPBS). Cell lysate was prepared using 200-μl volumes of lysis buffer (0.5% Triton-X100 in DPBS) which was repeatedly rinsed over the surfaces using a pipette before overnight incubation at 4°C to allow complete penetration of the detergent and solubilisation of the biological material. The total amount of DNA in cultures was measured using the Quant-iT™ PicoGreen® dsDNA kit calibrated with known concentrations of *λ* double-stranded DNA as previously described [18]. Fluorescence from samples was measured at excitation: 484 nm/emission: 538 nm using a Fluoroskan I plate reader (MTX Lab Systems, Inc., Vienna, Virginia, USA). DNA was measured 24 h post-seeding and at days 7, 14, 21 and 28. Data is presented as *N* = 3 biological replicate with each biological replicate taken as the average of three technical replicates.

#### Cell viability

Ti discs and Ti screws were transferred to empty wells of the 24-well cell culture cluster plate before a 4-h culture in a 500-μl volume of alamarBlue® (stock alamarBlue® reagent diluted by 1:10 in osteogenic medium; as previously described [18]. Equivalent reactions of Ti discs and Ti screws without cells were assembled in parallel to serve as a negative control. Medium samples (100-μl volumes) were transferred in triplicate into a 96-well plate, and the fluorescence read at excitation 544 nm/emission 585 nm using a Fluoroskan I plate reader. The results were normalised to the amount of DNA per square centimetre of the surface area. Cell viability was measured 24 h post-seeding and at days 7, 14, 21 and 28. Data is presented as *N* = 3 biological replicates with each biological replicate taken as the average of three technical replicates.

#### Mineralised matrix deposition

Ti discs and Ti screws were transferred to empty wells of the 24-well cell cluster plate before a 2-h incubation in a 500-μl volume of alizarin red (2% (*w*/*v*) alizarin red in ddH_2_O; pH 4.2). Ti discs and Ti screws were rinsed extensively with DPBS until the DPBS runoff was clear. Matrix was solubilised by incubating the Ti discs and Ti screws overnight in 200-μl volumes of lysis buffer (0.5% Triton-X100 in DPBS) at 4°C to allow complete penetration of the detergent and solubilisation of the biological material. Equivalent staining reactions of Ti discs and Ti screws without cells were assembled in parallel to serve as a negative control. Lysate samples (50-μl volumes) were transferred in triplicate into a 96-well plate and the absorbance read at A_492_ using a Multiscan Ascent plate reader (MTX Lab Systems Inc.). Mineralised matrix deposition was measured 24 h post-seeding and at days 7, 14, 21 and 28. Data is presented as *N* = 3 biological replicate with each biological replicate taken as the average of three technical replicates.

#### Statistical analysis

Statistical analyses were performed using the SPSS Statistics 19 work package (IBM). Data sets were evaluated using the Kolmogorov-Smirnov test to determine distribution. Data sets that were normally distributed were analysed by parametric Student *t*-test. The non-parametric equivalent Kruskal-Wallis *H* test was used for data that was not normally distributed.

## Results

**Figure 1:**
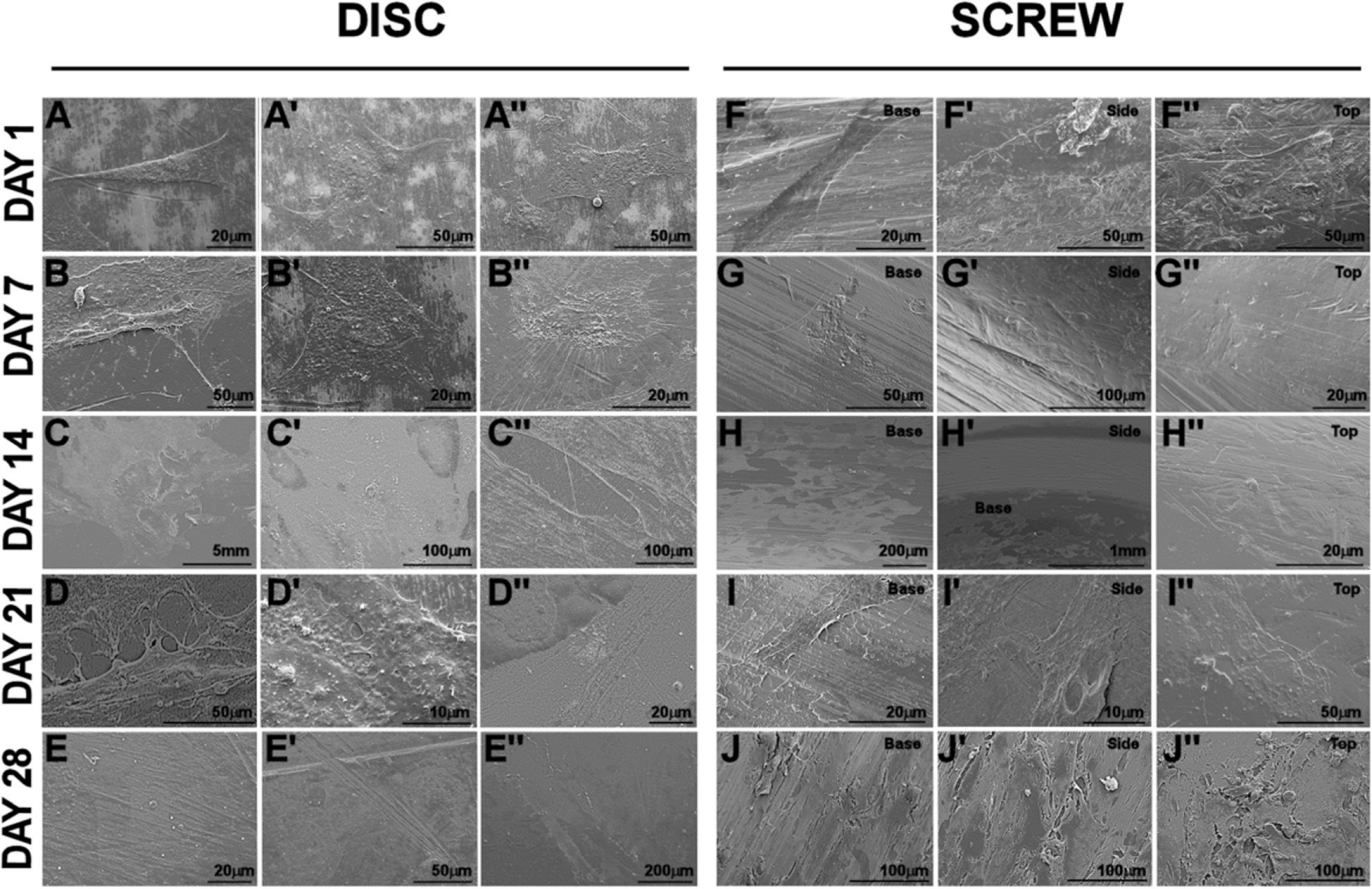
**Electron microscopy of HF-hMSC populations during osteogenic culture on titanium discs and screws.** HF-hMSC populations were seeded onto either Ti discs **(A-E)** or Ti screws **(F-J)** and cultured for up to 28 days in osteogenic medium. Representative images of three fields of view are presented for each time point at days 1, 7, 14, 21 and 28 post-seeding. Note at day 14, the restricted localisation of the cells to the core surface of the screw during expansion of the population.

HF-hMSCs were seeded onto Ti discs and Ti screws and cultured in osteogenic medium for up to 28 days. Electron microscopy analysis showed attachment and spreading of HF-hMSCs to Ti discs at day 1 with progressive expansion of the cell population across the surface of the disc (Figure 1A–B”). From day 14 of culture individual cells could not be discerned as electron micrographs revealed the progressive deposition of matrix (Figure 1C–E”). Whilst HF-hMSCs were also seen to attach and spread along the surface of the Ti screws, these cells were at first localised to the core surface at the base of the screw pitch (Figure 1F–G”). During osteogenic culture, the proliferation and expansion of the cell population was shown to be restricted exclusively to this basal region of the screw (day 14 of culture; Figure 1H–H”) before outgrowth up the sides of the screw pitch observed at day 21. By day 28 of osteogenic culture, the basal, side and top surfaces of the screw were covered by the cell population and evidence of deposited matrix was observed (Figure 1J–J”).

**Figure 2:**
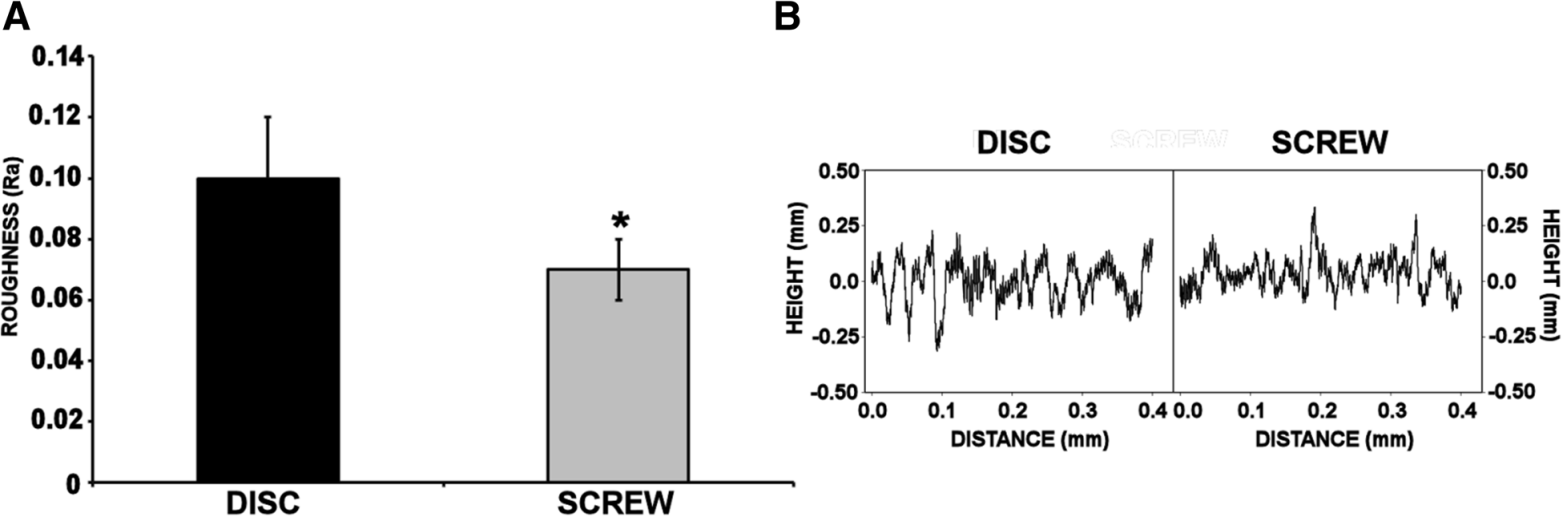
**Measurement of Ti surface roughness of disc and screw.** Roughness measurements were conducted using a stylus profilometer (Mitutoyo Surftest SV-2000 Mitutoyo, Halifax, UK) with dedicated analysis software (Surfpak- SV V1.600). **(A)** Average surface roughness analysed with a contact force of 4 mN and a maximum height range of 800 μm and an evaluation length of 0.4 mm. Values represent means ± S.E.M; *N* = 5; normalised data analysed by Student *t*-test; asterisk denotes *P* < 0.0007. **(B)** Example of a trace generated for the Ti disc and Ti screw.

Analysis of the roughness profiles for the disc and screw specimen revealed that the general form of the surfaces was similar. However, the roughness data revealed that the disc was significantly rougher than the screw (*t*-test, disc mean (SD) Ra 0.10 (0.02), screw mean (SD) Ra 0.07 (0.01), *P* = 0.0007) (Figure 2).

**Figure 3:**
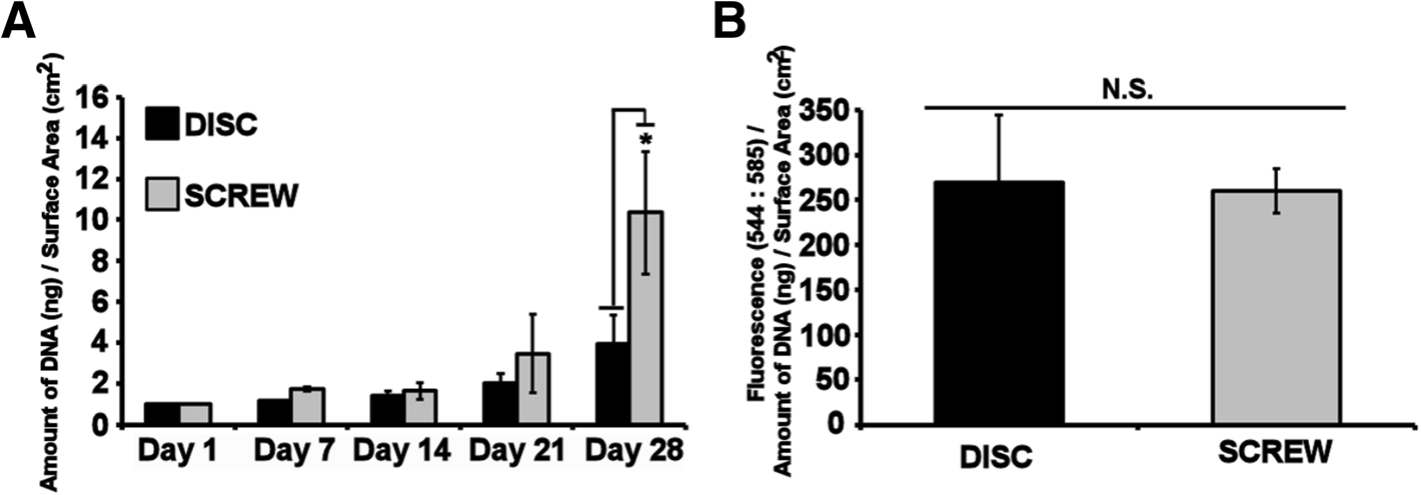
**Analysis of DNA content and cell vitality of HF-hMSC populations during osteogenic culture on titanium discs and screws.** HF-hMSC populations were seeded onto Ti discs and Ti screws and cultured for up to 28 days. **(A)** The amount of DNA within cultures was analysed using Picogreen® as a measure of cellular content, and results are presented as amount of DNA normalised to the surface area of the disc or screw. **(B)** Metabolic activity was analysed at day 28 using alamarBlue® as a measure of cellular vitality/health. Results are presented as resorufin fluorescence (Ex: 544/Em: 585) normalised to the amount of DNA per surface area of the disc or screw. Values represent means ± S.E.M; Data sets were not normally distributed and analysed by Kruskal-Wallis *H* test; *N* = 3 biological replicates where the average of each biological replicate was performed in technical replicates of three; asterisk denotes *P* < 0.05; N.S.: not significant.

Quantification of the cell content of Ti disc and Ti screw cultures was performed by quantification of DNA content during osteogenic culture and is presented as fold change in amount of DNA from day 1 per square centimetre of the surface area. Figure 3A shows a modest increase in DNA content on Ti discs with an approximate doubling of the cell population after 21 days of osteogenic culture. By day 28, the cell population had doubled again reflecting a fourfold increase in DNA content from day 1 of culture, representative of two rounds of cell division. The DNA content of osteogenic cultures on Ti screws was comparable with those of Ti discs at days 7 and 14; however, there was an increase in the amount of DNA between Ti screw and Ti disc cultures at day 21 (increase of 1.7 times; though not statistically significant) and at day 28 (increase of 2.6 times; *P* < 0.05). Overall there was a tenfold increase in the amount of DNA on Ti screws cultured over 28 days, representative of 3.125 cell divisions.

Because of the significant increase in cellular content on Ti screw cultures, a comparison of cellular vitality (cellular health) between osteogenic cell cultures on Ti discs and Ti screws was performed at day 28. Results are presented as measured fluorescence of alamarBlue® normalised to the amount of DNA per square centimetre of surface area and showed that there is no significant difference in cell vitality between the two culture surfaces (Figure 3B). This would indicate that the increase in cell number observed was a result of a positive increase in cell number (i.e. greater cell proliferation) rather than a loss of cells within the Ti disc cultures.

**Figure 4:**
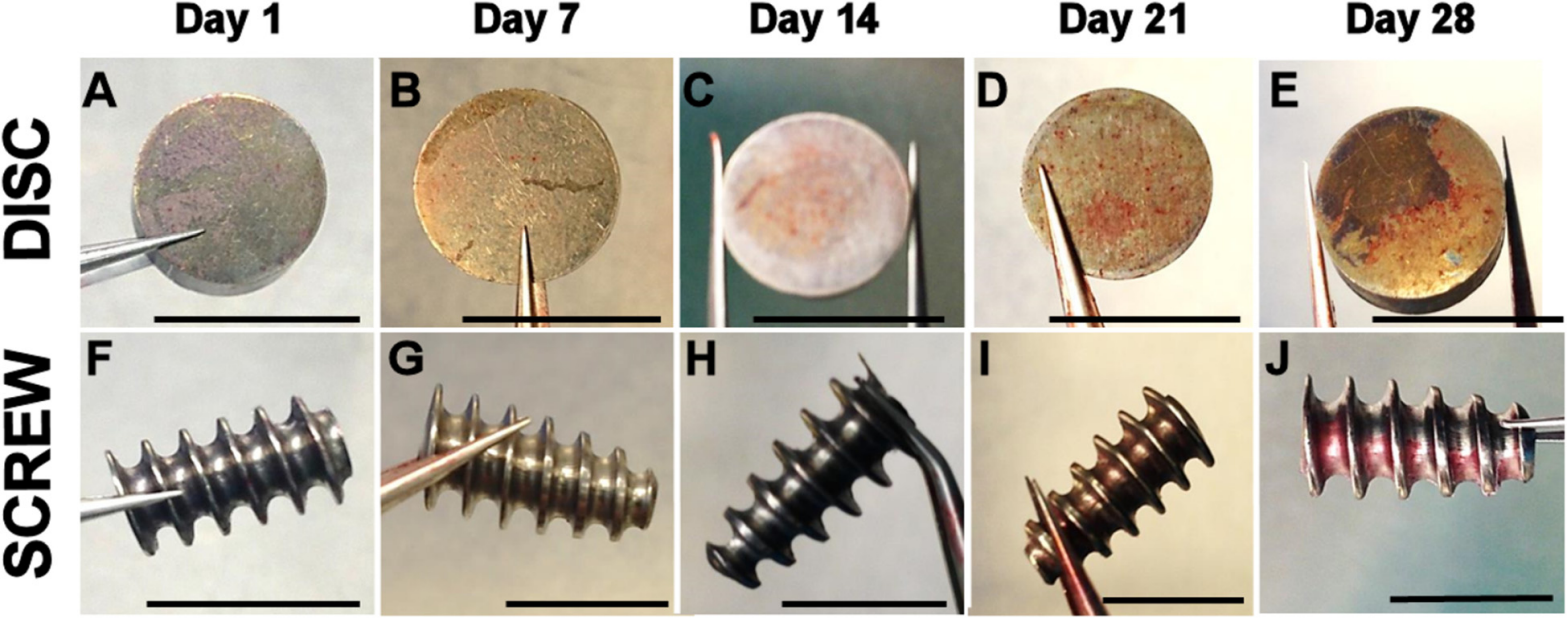
**Analysis of alizarin red staining during osteogenic culture of HF-hMSCs on Ti discs and Ti screws.** HF-hMSC populations were seeded onto Ti discs or Ti screws and cultured for up to 28 days in osteogenic medium. Matrix mineralisation on Ti surfaces was assessed by staining with alizarin red at days 1, 7, 14, 21 and 28 post-seeding. Scale bars = 1 cm.

**Figure 5:**
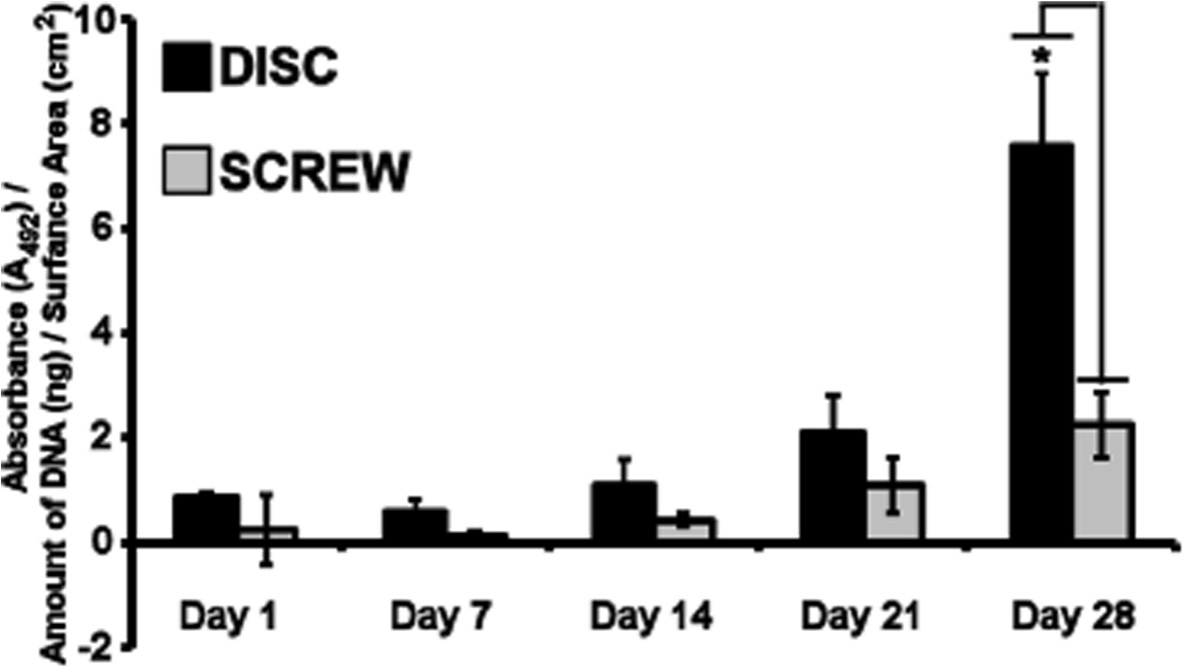
**Quantification of alizarin red staining during osteogenic cultures of HF-hMSCs on Ti discs and Ti screws.** HF-hMSC populations were seeded onto Ti discs or Ti screws and cultured for up to 28 days in osteogenic medium. Matrix, which had been stained with alizarin red, was solubilised. Quantification results are presented as the absorbance at A_492_ normalised to the amount of DNA per surface area of the Ti disc or Ti screw. Values represent means ± S.E.M; Data sets were not normally distributed and analysed by Kruskal-Wallis *H* test; *N* = 3 biological replicates where the average of each biological replicate was performed in technical replicates of three; asterisk denotes *P* < 0.05.

With evidence from the electron micrographs of the matrix on the surfaces of the titanium materials, we investigated the deposition of the mineralised matrix using alizarin red staining (Figure 4). Osteogenic cell cultures on Ti discs were negative of alizarin red staining at days 1 and 7. At day 14 of culture, modest alizarin red staining was observed and this was seen to become more intense as the osteogenic culture progressed to day 28 (Figure 4A–E). Osteogenic cultures on Ti screws were also negative for alizarin staining at days 1, 7 and 14; some alizarin red staining was evident at day 21 and this was intensified at day 28. Quantification of alizarin red staining was performed by measuring the absorbance of solubilised matrix (A_492_) normalised to the amount of DNA per square centimetre of the surface area and presented as fold increase from day 1 (Figure 5). Osteogenic cultures on Ti discs showed an increase in measured absorbance at day 21 (increase of 2.5 times; not statistically significant) and day 28 (increase of 9 times; *P* < 0.05). An increase in absorbance was also recorded for osteogenic cultures on Ti screws; however, after 28 days of osteogenic culture, this was only 30% of that recorded for those on Ti discs (*P* < 0.05).

## Discussion

Ti is frequently considered as the metal of choice for the fabrication of implants used in reconstructive surgeries because of its properties of biocompatibility and the low propensity to cause hypersensitivity. Further modification to the chemistry of the metal is often sought however as a means of enhancing the desirable characteristics required in its intended use. For instance, Ti used in the manufacture of screws for attaching bone to bone or soft tissue to bone can be mixed with other metals for example copper, nickel, cobalt or chronium to form an alloy with increased shear strength and the ability to withstand higher biomechanical loads [23]. Similarly, coating the surface layer has been shown to enhance the osteoconductive properties of the metal (e.g. strontium-incorporated Ti oxide, magnesium, calcium phosphate), improving graft integration within the host tissue [24-29].

Recent clinical examples have demonstrated how the poor choice of material used in the manufacture of medical devices can lead to adverse effects on the patient [30-32]. This has highlighted the need for greater understanding of the biological interactions that take place when developing materials to be used in advanced surgical technologies, particularly in order to meet the requirements of what most likely will be a tighter regulatory framework [30-32]. We are investigating a cellular approach to enhancing soft tissue graft integration with bone using patient-derived HF-hMSCs and in this study carried out a preliminary investigation of these cells in response to a standard, Ti interference screw which is increasingly used clinically for ACL repair particularly when undertaking revision surgeries [33,34]. Previous reports have demonstrated the *in vitro* attachment of MSCs to Ti surfaces, and this is confirmed by our electron micrograph data [24,35]. Of particular interest was the pattern of cell attachment on the Ti screw where culture expansion was seen to be restricted in the early stages to the base of the screw. Whilst the cells would have most likely settled at the screw base upon initial seeding of the HF-hMSC culture, there was a clear segregation between the base and the side of the screw, and it was only in the later stages of culture that cell expansion and matrix deposition was observed on the inner sides and the top of the screw.

Consistent with our previous findings of HF-hMSCs cultured on the surfaces of PLGA microparticles, cell culture expansion (measured by DNA content) was initially slow for both Ti discs and Ti screws [19]. This phenomenon has also been reported by Wall et al. [35] investigating the interaction of bone marrow-derived hMSCS with Ti surfaces. In this study, it was proposed that the Ti surface was selective for a particular population of cells within a heterogeneous mix of MSCs and these cells underwent a lag phase prior to becoming established on the Ti surface. This is certainly possible; it is known for instance that the tissue from which MSCs are derived can influence MSC behaviour on Ti surfaces. MSCs originating from ligamentous tissues having a greater attachment to smooth rather than rough surfaces compared to osteoblastic cells, which preferentially adhere to rough surfaces [35-37]. Within an *in vitro* study such as ours however, it may be assumed that the starting hMSC population has become relatively homogeneous through the process of derivation and culture expansion. We would therefore suggest an alternative theory for exploration in that the hMSCs require this lag phase in order to regulate the expression of different receptor proteins (e.g. integrins, cell surface proteoglycans) and extracellular matrix proteins (e.g. fibronectin, collagens) that allows for their adaptation to a new culture substrate; the influence of integrin regulation on osteogenic differentiation of hMSCs has previously been reported [38]. We did observe significant differences in the behaviour of cells cultured on control Ti discs and the Ti screws with greater cell content and less alizarin red-positive matrix recorded for the latter cultures. This finding may relate to the Ti screw population having a reduced differentiation response compared to the Ti disc cultures. For instance, the basal region of the screw may establish a microenvironment that influences HF-hMSCs. An example of this might be differences in oxygen tension; we have previously shown that oxygen tension influences the proliferation and differentiation of HF-hMSCs [18]. Alternatively there could be an increased concentration of paracrine factors that regulate differentiation. Previously, Uchida et al. [39] used a murine fracture model to demonstrate *in vivo* bone engraftment and showed that, whilst ossification occurs around the Ti screw, healing was different to that observed within other regions of graft attachment. The authors of this study concluded that ossification was directed by different MSC populations originating from the bone marrow that had been penetrated by the Ti screw; however, there may be contributing influences brought by the microenvironment of the screw.

In conclusion, our study has demonstrated that compatibility of HF-hMSCs with Ti screws may facilitate the retention of the HF-hMSCs at the site of surgery. The behaviour of the cells may be influenced by the shape of the implant, and this could create a complex biological environment that influences the behaviour of the HF-hMSCs.

## Abbreviations

ACL: anterior cruciate ligament
αMEM: alpha minimum essential medium
BMP2: bone morphogenetic protein-2
DPBS: Dulbecco’s phosphate-buffered saline
FBS: foetal bovine serum
FGF2: fibroblast growth factor-2
HF: haemarthrosis fluid
HF-hMSC: heamarthrosis fluid-derived human mesenchymal stem cell
hMSC: human mesenchymal stem cell
PCL: poly-ε-caprolactone
PLGA: poly(lactic-co-glycolic acid)
RCI: round cannulated interference
Ti: titanium
